# Delayed‐Onset Hepatocellular Liver Injury Associated With Nitrofurantoin and Herbal Supplement Use: A Case Report With Multiple Potential Exposures

**DOI:** 10.1155/crgm/1187155

**Published:** 2026-06-19

**Authors:** Edwin Mendoza, Manas Pustake, Jeffrey Annabi, Joshua Torres, Joanna Mendez, Lakshmi Kattamuri, Benjamin Williams, Abhizith Deoker

**Affiliations:** ^1^ Department of Internal Medicine, Texas Tech University Health Sciences Center El Paso, El Paso, Texas, USA, ttuhsc.edu; ^2^ Paul L. Foster School of Medicine, Texas Tech University Health Sciences Center El Paso, El Paso, Texas, USA, ttuhsc.edu; ^3^ Department of Pharmacy, University Medical Center El Paso, El Paso, Texas, USA; ^4^ Department of Pathology, Texas Tech University Health Sciences Center El Paso, El Paso, Texas, USA, ttuhsc.edu

**Keywords:** aflatoxin, autoimmune hepatitis mimic, Burnjaro, case report, drug-induced liver injury, liver biopsy, N-acetylcysteine, nitrofurantoin, weight-loss supplements

## Abstract

Nitrofurantoin, a first‐line antibiotic, works effectively for treating uncomplicated urinary tract infections, but it can cause severe liver damage. In the setting of concurrent supplement use, distinguishing what caused liver damage is challenging. A 57‐year‐old woman who had nonalcoholic fatty liver disease developed sudden jaundice after finishing her short nitrofurantoin treatment while consuming a commercial weight‐loss supplement. The laboratory results demonstrated a significant liver cell damage pattern with autoimmune test results positive while liver biopsy showed nonspecific inflammation that supported drug‐induced liver injury over drug‐induced autoimmune such as hepatitis. The patient received intravenous N‐acetylcysteine emergently after removing all suspected hepatotoxic substances from her treatment plan. N‐acetylcysteine was administered empirically due to concern for progression, although its role in nonacetaminophen DILI remains uncertain. The case demonstrates that clinicians should evaluate for polysubstance‐induced liver damage when patients present with multiple possible liver‐damaging substances.

## 1. Introduction

Nitrofurantoin is a commonly prescribed antimicrobial agent for the treatment and prophylaxis of uncomplicated urinary tract infections, valued for its efficacy, low resistance rates, and high urinary concentration. Nitrofurantoin shows excellent safety characteristics, but it can cause rare, dangerous liver problems which include acute and chronic hepatitis, cholestasis, granulomatous inflammation, autoimmune‐like reactions, and severe drug‐induced liver injury (DILI) [1, 2]. The exact causes of nitrofurantoin‐induced liver damage remain unknown, but it is thought to be from both immune system activation and tissue damage from free radicals [1, 2]. DILI accounts for a significant proportion of acute hepatitis cases approximately 33% per Galan et al., and establishing causality can be challenging, particularly when multiple potential hepatotoxins are involved [3]. The presence of autoimmune serologic markers further complicates the diagnostic process, as nitrofurantoin‐associated liver injury may mimic drug‐induced autoimmune‐like hepatitis (DI‐ALH) both clinically and histologically [4].

Aflatoxin, a potent hepatotoxin and carcinogen produced by Aspergillus species, represents another important cause of liver injury that can resemble drug‐ or toxin‐induced hepatitis. This toxin can be found in contaminated grains, nuts, spices, and even mushrooms which our patient was exposed to [5]. In the narrative review by Hamid et al., aflatoxin B1 is metabolized to a reactive epoxide that forms DNA adducts, promoting oxidative stress and mutagenesis which can lead to hepatocellular necrosis and jaundice [6]. Recognition of aflatoxin‐related injury is critical, particularly in patients with overlapping risk factors or ambiguous exposure histories. Burnjaro is an over‐the‐counter supplement known to have curcuma (or turmeric) and *Garcina cambogia*. Turmeric has been reported to cause hepatocellular injury as seen in 10 cases from a retrospective review of adjudicated cases enrolled in the DILI Network Prospective study between 2004 and March 2022 [7]. Turmeric can even mimic DI‐ALH, and removing this agent has been shown to cause self‐limiting injury in most cases [7, 8]. In a systemic review and retrospective study by Ballotin et al., subgroup analysis of 446 references demonstrated that *G. cambogia* was shown to cause mostly hepatocellular injury with recovery seen in one to 3 months [9]. However, in severe cases, liver transplantation was required [9]. In this study, we present a case of a 57‐year‐old woman who developed acute liver damage after taking nitrofurantoin, a commercial weight‐loss supplement, and eating wild mushrooms from the U.S.–Mexico border area, which made it difficult to identify the cause of her liver injury.

## 2. Case Presentation

A 57‐year‐old female with past medical history of nonalcoholic fatty liver disease (NAFLD), paroxysmal atrial fibrillation, hyperlipidemia, prediabetes, chronic hepatic cyst (biliary cystadenoma vs. minimally complex hepatic cyst), renal myolipoma, and small hepatic hemangioma presented with a 1‐week history of acute‐onset jaundice, involving the skin, sclera, face, chest, and abdomen, accompanied by dark urine. She reported a 1‐week history of constipation that resolved, associated with pale stools. She denied abdominal pain, fever, chills, nausea, vomiting, diarrhea, or initial pruritus. Family history was noncontributory for liver disease. In terms of social history, she is a never smoker and reports no current alcohol or illicit drug use. She is monogamous and had no recent travel or significant animal exposures.

Approximately a month before admission, she completed a 7‐day course of nitrofurantoin for cystitis. She also took an over‐the‐counter weight loss supplement (“Burnjaro”) for less than 30 days, discontinuing it at the same time of nitrofurantoin. Two weeks before admission, she ate mushrooms at a local restaurant in the US–Mexico border region, which she describes was white in color.

**FIGURE 1:**
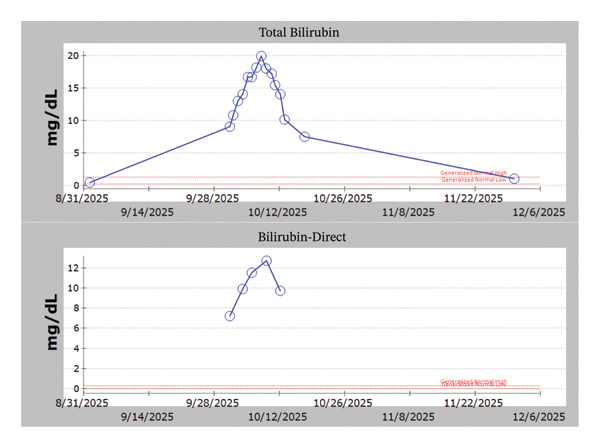
Bilirubin levels trends: top total bilirubin, bottom direct bilirubin.

On physical examination, the patient was alert, comfortable, and in no apparent distress. The most striking finding was profound scleral icterus and diffuse jaundice of her skin. Her abdominal examination was benign: soft, nontender, and without hepatosplenomegaly or palpable masses. Neurologically, she was intact with no signs of asterixis or encephalopathy. Initial laboratory investigations revealed a severe acute hepatocellular injury pattern, with transaminases in the thousands (AST 1767 U/L, ALT 2082 U/L) and a markedly elevated total bilirubin of 9.0 mg/dL, which was predominantly direct (Figure 1). Her calculated *R* index was 26.91, which was indicative of hepatocellular injury, while the RUCAM score was four indicating a possible likelihood of DILI [10]. Serum levels of IgG were within normal limits at 1350 mg/dL, which range from 600 to 1640 mg/dL. Her international normalized ratio (INR) was normal at 1.2, indicating preserved synthetic liver function.

**FIGURE 2:**
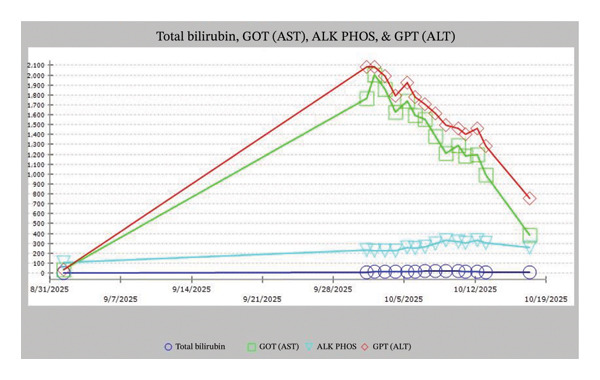
Laboratory trends.

A diagnostic workup was initiated to elucidate the etiology of her acute hepatitis. Imaging with right upper quadrant ultrasound, CT abdomen and pelvis, and MRCP ruled out biliary obstruction as a cause. The studies confirmed a known stable hepatic cyst and findings consistent with her background NAFLD, but showed no evidence of mass lesions or ductal dilation. An extensive serological panel was negative for acute viral hepatitis A, B, or C. Autoimmune workup returned a positive antinuclear antibody (ANA), raising an initial potential consideration of autoimmune hepatitis, though antismooth muscle antibody and anti‐dsDNA were negative. Further metabolic workup, including ceruloplasmin, was unremarkable. Figure 2 and Table 1 depict the bloodwork and laboratory trends.

**TABLE 1:** Diagnostic workup.

**Diagnostic Assessment**

Initial Laboratory Findings:
Liver Enzymes: Marked transaminitis (AST 1767 U/L, ALT 2082 U/L) with alkaline phosphatase (ALP) 230 U/L and GGT 270 U/L.
Bilirubin: Total bilirubin 9.0 mg/dL (direct 7.2 mg/dL), which subsequently peaked at 18.1 mg/dL.
Coagulation: INR 1.2 (stable).
Other: Mild hyponatremia, albumin 3.4 g/dL. Urinalysis was positive for large bilirubin.
Imaging:
RUQ Ultrasound and CT Abdomen/Pelvis: Contracted gallbladder with wall thickening and sludge; no cholelithiasis or biliary ductal dilation. Confirmed a stable 4.7–5.3 cm lobulated cyst in the left hepatic lobe, hepatic steatosis, and mild hepatomegaly.
MRCP/MRI Abdomen: No evidence of extrahepatic biliary obstruction. Stable hepatic cyst with mild segmental intrahepatic bile duct dilation posterior to the cyst.
Etiological Workup:
Infectious: Hepatitis A, B, C serologies negative. HIV negative.
Autoimmune: ANA positive (> 1:160). Anti‐dsDNA, antismooth muscle antibody, and IgG levels were negative or pending.
Metabolic/Toxic: Ceruloplasmin normal. Aflatoxin IgE level low 0.36 kU/L (0.35–0.69 kU/L low level). Iron studies showed elevated ferritin but were not diagnostic for hemochromatosis.
Liver Biopsy:
A percutaneous liver biopsy was performed. Histopathological examination revealed nonspecific acute hepatitis with an inflammation‐predominant pattern. The findings were deemed most consistent with a drug‐related acute hepatitis. The pathology report noted that while both nitrofurantoin and aflatoxin can cause injury, the inflammation‐predominant pattern in this case favored DILI, and the progression of injury after drug cessation is a recognized feature of nitrofurantoin toxicity.

## 3. Differential Diagnosis

The primary differential diagnoses on admission were as follows:

1. DILI was secondary to recent nitrofurantoin use and/or the “Burnjaro” supplement.
2. DI‐ALH was considered due to positive ANA results.
3. Aflatoxin toxicity was initially considered but deemed less likely based on history and preliminary biopsy findings.
4. Other causes (viral, obstructive, Wilson disease, and Alpha‐1 antitrypsin deficiency) were ruled out.

Given the diagnostic dilemma between DILI, DI‐ALH, and the less likely possibility of mycotoxin exposure from a contaminated supplement, a percutaneous liver biopsy was performed. The histopathological analysis revealed a pattern of nonspecific acute hepatitis that was inflammation‐predominant. This morphology was most consistent with a drug‐induced etiology. While both nitrofurantoin and aflatoxin (a potential contaminant) can cause liver injury, the inflammation‐predominant pattern, in this case, favored DILI. Aflatoxin IgE was ordered which demonstrated low levels, 0.36 kU/L (0.35–0.69 kU/L low level). Although aflatoxin IgE testing was obtained, this is not a validated marker for aflatoxin‐induced hepatotoxicity and was not used to guide clinical decision‐making. What should have been ordered were aflatoxin metabolites or adducts, which were not performed in this case. In the online book chapter by Dhakal et al., detection of aflatoxin toxicity involves both direct measurements of food or metabolites in the body [11]. Although these methods were not used to further investigate aflatoxin toxicity, nevertheless, biopsy results did not show classic histopathology seen in aflatoxin hepatoxicity such as acute hemorrhagic necrosis, fatty changes in hepatocytes, or bile duct proliferation.

### 3.1. Therapeutic Intervention and Hospital Course

The patient’s management was primarily supportive, along with N‐acetylcysteine (NAC), centered on the withdrawal of all potential hepatotoxins. Due to the patient’s persistently elevated liver enzymes (Day 1 AST 1767 units/L with Day 2 AST 2006 and Day 1 ALT 2083 units/L with Day 2 ALT 2082 units/L) when she was admitted along with jaundice, given the risk of progression to acute liver failure, a joint decision was made without full liver workup completed to give NAC. Although most DILI improves after removal of the offending agent, patients with hepatocellular jaundice have a high mortality rate, which as indicated by Björnsson et al., AST and bilirubin levels are important independent predictors of death or liver transplantation [12]. Therefore, although NAC is not indicated in nonacetaminophen DILI in the absence of acute liver failure, the up‐trending liver function tests from Day 1 to Day 2 raised concerns of impending liver failure, and NAC was given. While a randomized controlled trial by Lee et al. demonstrated improved transplant‐free survival in early‐stage nonacetaminophen acute liver failure, this benefit was limited to patients with hepatic encephalopathy, and its role in uncomplicated DILI remains unclear [13]. The gastroenterology consult team withheld corticosteroid treatment, as the biopsy strongly favored DILI over autoimmune hepatitis, and there was no evidence of clinical deterioration [14]. Throughout her 2‐week hospitalization, she remained hemodynamically stable and mentally intact, showing no signs of progression to acute liver failure. Her liver enzymes began a steady downward trend, and her bilirubin, after peaking at 18.1 mg/dL, also commenced a slow decline.

### 3.2. Final Diagnosis and Outcome

The final diagnosis was moderate acute hepatocellular DILI, deemed most likely secondary to nitrofurantoin, and “Burnjaro” supplement. The positive autoimmune serology was considered an epiphenomenon rather than the primary driver of her liver injury. By the time of discharge, the patient was clinically stable with improving laboratory markers. She was discharged with close outpatient follow‐up arranged with gastroenterology for continued monitoring of her liver function and with urology for surveillance of her known renal angiomyolipomas.

## 4. Discussion

This case highlights the diagnostic complexity of polysubstance‐induced hepatotoxicity, particularly when autoimmune markers are present or multiple potential hepatotoxins such as nitrofurantoin and Burnjaro are involved. Although herbal and dietary supplements have been increasingly associated with hepatotoxicity, the temporal relationship and histologic pattern in this case strongly supported nitrofurantoin and Burnjaro as most likely contributors.

Nitrofurantoin is a commonly used antibiotic recommended as first‐line therapy in uncomplicated cystitis due to its high urinary concentration, clinical efficacy, and general tolerability [1]. However, rare but serious side effects such as pulmonary toxicity and DILIs have been reported. Acute injury is exceedingly rare (∼0.3 cases per 100,000 prescriptions) and typically arises a few weeks after starting or stopping treatment typically following a hepatotoxic injury profile [4]. Chronic injury is more common (between 1 per 1500 and 1 per 3000) and typically presents months to years after induction of long‐term prophylactic therapy [4]. In this case, our patient developed severe hepatocellular injury approximately 1 month after completing a short course, which is consistent with previously described acute presentations.

Features of autoimmune hepatitis have been described in multiple studies, often with long‐term exposure to nitrofurantoin but can also be seen with short duration of use [15]. In a prospective cohort study of 88 patients from the DILI Network data, the onset of liver injury varied from weeks to years after exposure [2]. Risk factors for DILI include older age, female gender, and chronic use of nitrofurantoin > 6 months for urinary tract infection prevention [1, 2]. Garcia‐Cortes et al. in an observational, descriptive analysis of two prospective DILI registries of 1426 cases of DILI, only 2.3% had autoimmune features [16]. The patient’s Naranjo adverse drug reaction probability scale scored 4 out of 13, meaning it was possible for nitrofurantoin to be the cause of her liver injury (Table 2) [17, 18]. Our patient presented with severe acute hepatocellular injury approximately 1 month after completing a short course of nitrofurantoin, with concurrent exposure to a weight‐loss supplement containing ingredients previously associated with hepatotoxicity. At least two of the listed ingredients within “Burnjaro” have sufficient evidence published correlating to liver injury. The overall clinical picture suggests a likely contribution from both nitrofurantoin and Burnjaro.

**TABLE 2:** Naranjo adverse drug reaction probability scale^6^ [1].

	**Answer**	**Score**

1) Are there previous conclusive reports on this reaction?	Yes	+1
2) Did the adverse event appear after the suspected drug was administered?	Yes	+2
3) Did the adverse reaction improve when the drug was discontinued or a specific antagonist was administered?	Yes	+1
4) Did the adverse reaction reappear when the drug was readministered?	Do not know	0
5) Are there alternative causes (other than the drug) that could on their own have caused the reaction?	Yes	−1
6) Did the reaction reappear when a placebo was given?	Do not know	0
7) Was the drug detected in the blood (or other fluids) in concentrations known to be toxic?	Do not know	0
8) Was the reaction more severe when the dose was increased, or less severe when the dose was decreased?	Do not know	0
9) Did the patient have a similar reaction to the same or similar drugs in *any* previous exposure?	Do not know	0
10) Was the adverse event confirmed by any objective evidence?	Yes	+1
Total		4

DILI is the diagnosis of exclusion requiring a detailed medication history, unraveling characteristic laboratory findings, considering other differential diagnosis, and assessment of the temporal relationship between drug exposure and liver injury [13]. Tools such as RUCAM provide objective support when exposure history or serological testing is inconclusive [10]. As discussed by Navarro et al., herbal and dietary‐induced liver injury now makes up 20% of the cases of hepatotoxicity in the United States [15, 19].

A key challenge in this case was distinguishing DILI from DI‐ALH. Although ANA was positive, other features required to support this diagnosis were absent. Lab work demonstrated that levels of IgG serum were within normal limits at 1350 mg/dL ranging from 600 to 1640 mg/dL. Alkaline phosphatase was 230 unit/L (35–104 unit/L), and GGT was 270 unit/L (5–36 unit/L). The liver biopsy did not mention interface hepatitis but rather had acute, likely resolving hepatitis. The *R* factor was 26.91 which is indicative of hepatocellular injury, while the RUCAM score is four indicating a possible likelihood of DILI [10]. Viral infections such as hepatitis *E* should also be screened for when patients are admitted, which our patient unfortunately did not undergo testing for it [20]. In addition, the patient improved without corticosteroid therapy. These findings support a diagnosis of DILI with autoimmune features rather than true DI‐ALH. Nevertheless, it is critical to distinguish DI‐ALH from autoimmune‐like hepatitis (AIH), as DI‐ALH often resolves with removing the offending medication and does not require long‐term immunosuppression therapy [21].

Management is centered on discontinuation of all potential hepatotoxic agents and supportive care. NAC is not indicated for the treatment of other etiologies of DILI besides acetaminophen toxicity, and evidence for utilizing NAC for aflatoxin‐related acute liver injury is limited to animal studies [22, 23].

**FIGURE 3:**
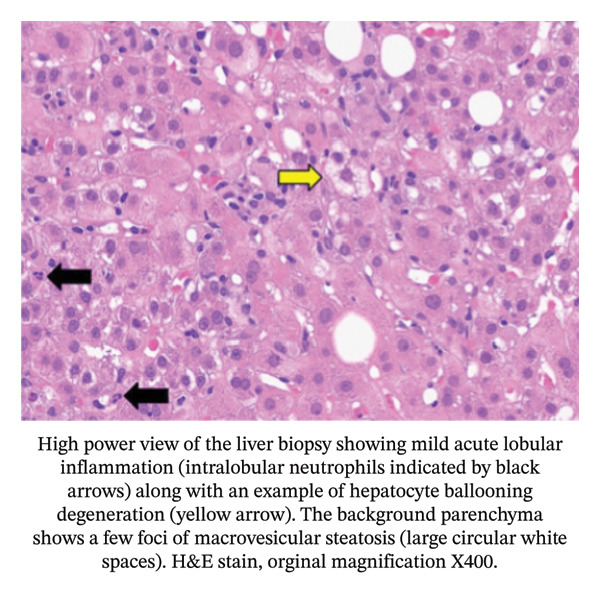
Liver biopsy pathology results: high power view of the liver biopsy showing mild acute lobular inflammation (intralobular neutrophils indicated by black arrows) along with an example of hepatocyte ballooning degeneration (yellow arrow). The background parenchyma shows a few foci of macrovesicular steatosis (large circular white spaces). H&E stain, original magnification X400.

**FIGURE 4:**
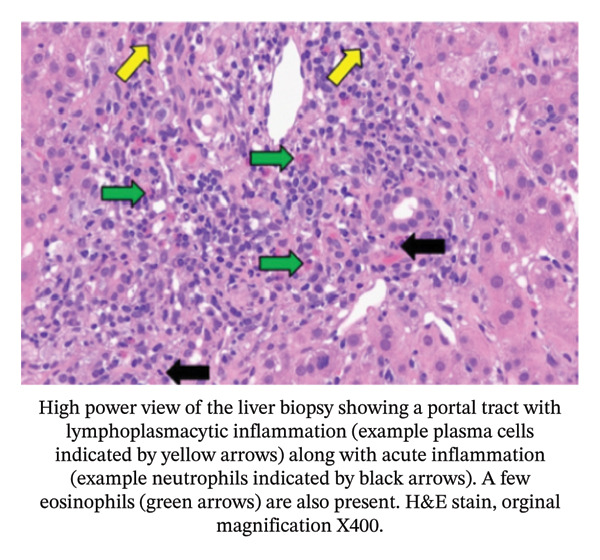
Liver biopsy pathology results: high power view of the liver biopsy showing a portal tract with lymphoplasmacytic inflammation (example plasma cells indicated by yellow arrows) along with acute inflammation (for example, neutrophils indicated by black arrows). A few eosinophils (green arrows) are also present. H&E stain, original magnification X400.

**FIGURE 5:**
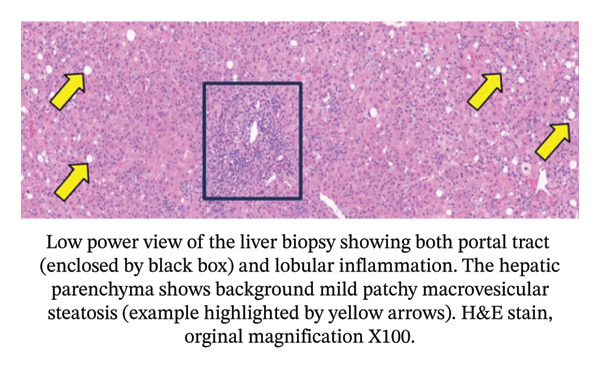
Liver biopsy pathology results: low power view of the liver biopsy showing both portal tract (enclosed by black box) and lobular inflammation. The hepatic parenchyma shows background mild patchy macrovesicular steatosis (for example, highlighted by yellow arrows). H&E stain, original magnification X400.

This case emphasizes the importance of maintaining a high index of suspicion for polysubstance‐induced hepatotoxicity, even after short‐term therapeutic courses. The onset and features described in the literature, along with our patient’s qualifying risk factors, strongly suggest both nitrofurantoin and Burnjaro as the causes of her severe acute hepatocellular liver injury. Additionally, biopsy results illustrate that this pattern of liver injury favors DILI (Figures 3, 4, 5). Clinicians should be aware that this complication can occur even with standard short course therapy, and presentation may be delayed by several weeks following drug discontinuation. Early recognition, careful mediation reconciliation, prompt withdrawal of the offending agent, and supportive management remain crucial for favorable outcomes in cases of DILI.

## Author Contributions

Edwin Mendoza, Manas Pustake, Jeffrey Annabi, Joshua Torres, Joanna Mendez, and Lakshmi Kattamuri contributed to the conception of the work, data collection, and manuscript drafting. Abhizith Deoker assisted with data interpretation, manuscript revision, and provided clinical oversight and critical revisions. Benjamin Williams contributed to pathological interpretation and review of histologic findings.

## Funding

No funding was received for this work.

## Disclosure

All authors reviewed and approved the final version of the manuscript.

## Ethics Statement

This case report was conducted in accordance with the institutional and ethical standards. Written informed consent was obtained from the patient for publication of this case and the accompanying images. All efforts were made to protect patient confidentiality, and no identifying information is included in the manuscript.

## Consent

Please see the Ethics Statement.

## Conflicts of Interest

The authors declare no conflicts of interest.

## Data Availability

All relevant data supporting the findings of this case are included within the manuscript. Additional details are not publicly available to preserve patient confidentiality but may be provided by the corresponding author upon reasonable request.

## References

[1] LiverTox and Clinical and Research Information on Drug-Induced Liver Injury , Bethesda (MD): National Institute of Diabetes and Digestive and Kidney Diseases, 2012, Nitrofurantoin, https://www.ncbi.nlm.nih.gov/books/NBK548318/. 31643176

[2] Sakaan S. A. , Twilla J. D. , Usery J. B. , Winton J. C. , and Self T. H. , Nitrofurantoin-Induced Hepatotoxicity: A Rare Yet Serious Complication, Southern Medical Journal. (2015) 107, no. 2, 107–113, 10.1097/SMJ.0000000000000059. 24926677

[3] Galan M. V. , Potts J. A. , Silverman A. L. , and Gordon S. C. , The Burden of Acute Nonfulminant Drug-Induced Hepatitis in a United States Tertiary Referral Center, Journal of Clinical Gastroenterology. (2005) 39, no. 1, 64–67, 10.1097/mcg.0000148380.81683.74. 15599214

[4] Chalasani , Li Y. J. , Dellinger A. et al., Clinical Features, Outcomes and HLA Risk Factors Associated With Nitrofurantoin-Induced Liver Injury, Journal of Hepatology. (2023) 78, no. 2, 293–300, 10.1016/j.jhep.2022.09.010. 36152763 PMC9852026

[5] Shabeer S. , Asad S. , Jamal A. , and Ali A. , Aflatoxin Contamination, Its Impact and Management Strategies: An Updated Review, Toxins. (2022) 14, no. 5, 10.3390/toxins14050307. PMC9147583 35622554

[6] Hamid A. S. , Tesfamariam I. G. , Zhang Y. , and Zhang Z. G. , Aflatoxin B1-Induced Hepatocellular Carcinoma in Developing Countries: Geographical Distribution, Mechanism of Action and Prevention, Oncology Letters. (2013) 5, no. 4, 1087–1092, 10.3892/ol.2013.1169. 23599745 PMC3629261

[7] Halegoua-DeMarzio D. , Navarro V. , Ahmad J. et al., Liver Injury Associated With Turmeric-A Growing Problem: Ten Cases From the Drug-Induced Liver Injury Network [DILIN], Americas Journal of Medicine. (2023) 136, no. 2, 200–206, 10.1016/j.amjmed.2022.09.026. PMC9892270 36252717

[8] Lombardi N. , Crescioli G. , Maggini V. et al., Acute Liver Injury Following Turmeric Use in Tuscany: An Analysis of the Italian Phytovigilance Database and Systematic Review of Case Reports, British Journal of Clinical Pharmacology. (2021) 87, no. 3, 741–753, 10.1111/bcp.14460. 32656820

[9] Ballotin V. R. , Bigarella L. G. , Brandao A. B. M. , Balbinot R. A. , Balbinot S. S. , and Soldera J. , Herb-Induced Liver Injury: Systematic Review and Meta-Analysis, World Journal of Clinical Cases. (2021) 9, no. 20, 5490–5513, 10.12998/wjcc.v9.i20.5490. 34307603 PMC8281430

[10] Danan G. and Teschke R. , RUCAM in Drug and Herb Induced Liver Injury: the Update, International Journal of Molecular Sciences. (2015) 17, no. 1, 10.3390/ijms17010014. PMC4730261 26712744

[11] Dhakal A. , Hashmi M. F. , and Sbar E. , Aflatoxin Toxicity, 2026, StatPearls Publishing, https://www.ncbi.nlm.nih.gov/books/NBK557781/. 32491713

[12] Bjornsson E. and Olsson R. , Outcome and Prognostic Markers in Severe Drug-Induced Liver Disease, Hepatology. (2005) 42, no. 2, 481–489, 10.1002/hep.20800. 16025496

[13] Lee W. M. , Hynan L. S. , Rossaro L. et al., Intravenous N-Acetylcysteine Improves Transplant-Free Survival in Early Stage Non-Acetaminophen Acute Liver Failure, Gastroenterology. (2009) 137, no. 3, 856–864, 10.1053/j.gastro.2009.06.006. 19524577 PMC3189485

[14] Fontana R. J. , Liou I. , Reuben A. et al., AASLD Practice Guidance on Drug, Herbal, and Dietary Supplement-Induced Liver Injury, Hepatology. (2023) 77, no. 3, 1036–1065, 10.1002/hep.32689. 35899384 PMC9936988

[15] Boer Y. S. , Zhao Z. , Long N. et al., Features of Autoimmune Hepatitis in Patients With Drug Induced Liver Injury, Clinical Gastroenterology and Hepatology. (2017) 15, no. 1, 103–112, 10.1016/j.cgh.2016.05.043. 27311619 PMC5370577

[16] Garcia-Cortes M. , Ortega-Alonso A. , Matilla-Cabello G. et al., Clinical Presentation, Causative Drugs and Outcome of Patients With Autoimmune Features in Two Prospective DILI Registries, Liver International. (2023) 43, no. 8, 1749–1760, 10.1111/liv.15623. 37269163

[17] Luk T. , Edwards B. D. , Bates D. , Evernden C. , and Edwards J. , Nitrofurantoin-Induced Liver Failure: A Fatal Yet Forgotten Complication, Canadian Family Physician. (2021) 67, no. 5, 342–344, 10.46747/cfp.6705342. 33980626 PMC8115952

[18] Bjornsson E. S. , Clinical Management of Patients With Drug-Induced Liver Injury (DILI), United European Gastroenterology Journal. (2021) 9, no. 7, 781–786, 10.1002/ueg2.12113. 35084797 PMC8435256

[19] Navarro V. J. , Khan I. , Bjornsson E. , Seeff L. B. , Serrano J. , and Hoofnagle J. H. , Liver Injury From Herbal and Dietary Supplements, Hepatology. (2017) 65, no. 1, 363–373, 10.1002/hep.28813. 27677775 PMC5502701

[20] European Association for the Study of the Liver , Electronic Address eee , Clinical Practice Guidelines P , and Wendon J. , EASL Clinical Practical Guidelines on the Management of Acute (Fulminant) Liver Failure, Journal of Hepatology. (2017) 66, no. 5, 1047–1081, 10.1016/j.jhep.2016.12.003. 28417882

[21] Andrade R. J. , Aithal G. P. , de Boer Y. S. et al., Nomenclature, Diagnosis and Management of Drug-Induced Autoimmune-Like Hepatitis (DI-ALH): An Expert Opinion Meeting Report, Journal of Hepatology. (2023) 79, no. 3, 853–866, 10.1016/j.jhep.2023.04.033. 37164270 PMC10735171

[22] European Association for the Study of the L , EASL Clinical Practice Guidelines: Drug-Induced Liver Injury, Journal of Hepatology. (2019) 70, no. 6, 1222–1261, 10.1016/j.jhep.2019.02.014. 30926241

[23] Valdivia A. G. , Martinez A. , Damian F. J. et al., Efficacy of N-Acetylcysteine to Reduce the Effects of Aflatoxin B1 Intoxication in Broiler Chickens, Poultry Science. (2001) 80, no. 6, 727–734, 10.1093/ps/80.6.727. 11441839

[24] Naranjo C. A. , Busto U. , Sellers E. M. et al., A Method for Estimating the Probability of Adverse Drug Reactions, Clinical Pharmacology & Therapeutics. (1981) 30, no. 2, 239–245, 10.1038/clpt.1981.154. 7249508

[25] Bjornsson E. S. , Medina-Caliz I. , Andrade R. J. , and Lucena M. I. , Setting up Criteria for Drug-Induced Autoimmune-Like Hepatitis Through a Systematic Analysis of Published Reports, Hepatology Communications. (2022) 6, no. 8, 1895–1909, 10.1002/hep4.1959. 35596597 PMC9315110

